# Perceived determinants of the use of coercion in inpatient child and adolescent psychiatry: a qualitative interview study with staff

**DOI:** 10.1186/s12888-025-06690-x

**Published:** 2025-03-17

**Authors:** Astrid Moell, Alexander Rozental, Susanne Buchmayer, Riittakerttu Kaltiala, Niklas Långström

**Affiliations:** 1 Centre for Psychiatry Research, Department of Clinical Neuroscience, Karolinska Institutet, & Stockholm Health Care Services, Region Stockholm, Stockholm, Sweden; 2 Department of Health, Education and Technology, Luleå University of Technology, Luleå, Sweden; 3 Faculty of Medicine and Health Technology, Tampere University, Tampere, Finland; 4 Department of Adolescent Psychiatry, Tampere University Hospital, Tampere, Finland; 5 Vanha Vaasa Hospital, Vaasa, Finland

**Keywords:** Coercive measures, Child and adolescent mental health, Inpatient care, Psychiatry, Coercion, Informal coercion, Treatment pressures, Staff

## Abstract

**Background:**

Understanding factors influencing the use of coercive practices in clinical psychiatry is necessary to develop strategies to reduce their use. However, there is little evidence regarding staff perceptions of such factors, particularly in inpatient child and adolescent psychiatry (CAP).

**Methods:**

We conducted semi-structured interviews with nurses, senior consultants and heads of units in inpatient CAP in Sweden 2021 (N = 9). The interviews were transcribed verbatim and analysed using reflexive thematic analysis. Data on informal coercion were analysed separately using a deductive approach based on previously proposed hierarchies for informal coercion.

**Results:**

We identified one overarching theme of factors reported to influence the use of coercive practices: “Trust and distrust in coercive and non-coercive approaches”, in turn encompassing the two subthemes “Ward culture” and “Available resources and strain”. Our findings suggest a risk of a *negative spiral of coercion* emerging when there is low professional trust in non-coercive approaches and high trust in coercive methods. Informal coercion was used frequently and observed to occur in two distinct processes: one concerning continuous coercive escalation, and the other involving sustained efforts at the same coercion level.

**Conclusions:**

Trusting the efficacy of non-coercive approaches in inpatient CAP care appears critical for their success; a finding that may inform strategies to reduce coercion and address frequent use with individual patients.

**Supplementary Information:**

The online version contains supplementary material available at 10.1186/s12888-025-06690-x.

## Background

Coercive measures (i.e., mechanical and physical restraint, seclusion, and involuntary treatment including medication and forced tube-feeding) remain commonly used both in adult psychiatry [1] and in child and adolescent psychiatry (CAP) [2], despite considerable concerns regarding possible negative effects [3–5]. Coercive measures are usually applied to manage conflict (e.g., severe self-harm or aggression towards others) or to enforce patient adherence to treatment. There is a growing movement to reduce or even abolish coercion in mental health services; the Council of Europe has urged member states to abolish coercive practices [6], while the World Psychiatric Association calls for strategies that respect patient autonomy and promote non-coercive care [7]. Efforts to reduce the use of coercive measures include different multimodal approaches, often combining prevention strategies, staff training, patient-centred care, and organisational changes [8–11]. However, as these multicomponent interventions target both individual and structural factors, isolating the effects of specific components remains challenging [12].

*Formal coercion* refers to legally authorised interventions, including involuntary hospitalisation, and coercive measures. A dimension of coercion that remains less explored is *informal coercion*, sometimes referred to as “treatment pressures” [13]. Informal coercion lacks a comprehensive and universally accepted definition, but it refers to various non-legal strategies and interpersonal dynamics employed by healthcare professionals to influence treatment decisions [14]. The coercion hierarchy proposed by Szmukler and Appelbaum [13] outlines increasing levels of influence on patient decisions: from persuasion and interpersonal leverage (e.g., staff expressing disappointment about non-adherence) to inducements (e.g., offering incentives for treatment adherence), threats, and deception or strategic dishonesty. More recent additions to the conceptualisation of informal coercion include using a disciplinary style (e.g., enforcing ward rules through structured consequences, such as withholding food if the patient arrives late for dinner), referring to rules and routines, and coercion from other stakeholders [15]. Despite its frequent occurrence in clinical practice [16], informal coercion has not yet received the same scientific attention as formal coercion.

Systematic reviews of adult psychiatric staff attitudes towards coercive practices suggest that while coercive measure use is viewed as unavoidable and necessary at times, their administration is generally a negative experience for staff [17]. Moreover, a paradigm shift seems to have occurred; from perceiving coercive measures as therapeutic with beneficial effects for the patient to viewing them as undesirable but necessary for ensuring safety [18]. Another integrative review also found adult psychiatry nurses’ decision-making regarding coercive measures to be influenced by factors such as safety for all patients and staff, the perception of restraint as a necessary intervention but also to be used as a “last resort”, role conflicts, and their psychological impact on staff [19]. This review also highlighted the importance of staffing levels and staffing experience in the decision-making process. Further, a scoping review of both quantitative and qualitative studies from adult psychiatry indicated highly variable attitudes towards coercive practices, in part shaped by professional background, cultural context and work experience [20]. One previous qualitative study used ethical diaries to examine staff ethical considerations in inpatient CAP [21], indicating that participants rarely perceived formal coercion as problematic. However, some coercive measures were described as emotionally difficult to carry out. Apart from that study’s ethical perspective, no study to date has explored inpatient CAP staff understanding and attitudes towards coercive practices from a broader perspective. Exploring staff attitudes towards both formal and informal coercion is important to identify factors influencing its use; filling this knowledge gap could further inform the development of interventions to mitigate coercive practices.

### Study aims

We aimed to explore inpatient CAP staff perceptions, clinical use and perceived determinants of coercive measures and informal coercion.

## Methods

### Study design

We conducted a qualitative study on staff working in inpatient CAP in Sweden using semi-structured individual online video interviews.

### Setting

Swedish CAP care is publicly funded and governed by national legislation, with inpatient care provided at specialised psychiatric units, typically separate from adult psychiatric care. In 2023, 2789 individuals received inpatient CAP care in Sweden, accounting for 39,495 inpatient days, with a median length of stay of 8.7 days, an overall bed occupancy rate of 75%, and 20% of all admissions being compulsory. [22]. A survey across 28 European countries found that Sweden had the lowest inpatient CAP bed capacity per capita [23]. This limited capacity may contribute to increased work-related stress, with more than half of staff in a recent Swedish inpatient CAP study considering resigning at least a few times per month [24]. Further details, including the legislative framework, are provided in the supplementary material of our previous publication (supplementary material) [25].

### Participant recruitment and sampling

The inclusion criteria for participants required them to be working as nurses, senior consultants in child and adolescent psychiatry (psychiatrists), or heads of units in inpatient CAP units in Sweden for at least 6 months before and 6 months after a legal change regarding coercive measures on July 1, 2020. These occupational categories were selected because nurses implement coercive measures, senior consultants make the formal decisions, and heads of units oversee the overall practices within their units.

Participant recruitment was halted in October 2021 following a critical statement issued by the Swedish Chief Parliamentary Ombudsman regarding legal practices related to the use of coercive measures in CAP inpatient care [26]. This statement was considered likely to influence participants’ responses, potentially preventing them from accurately reflecting upon their current or prior practices.

We recruited participants through e-mails to all regional heads of CAP in Sweden on April 28, 2021 (a reminder email was sent on May 10), requesting dissemination to all staff in inpatient CAP (i.e., a recruitment process in qualitative research referred to as gatekeepers). Staff interested in participation were asked to contact the project coordinator AM. Twelve potential participants reached out to the coordinator; two were not included due to insufficient clinical experience, and a third was excluded due to ongoing scheduling conflicts for the interview. A total of nine participants were included, see Table 1 for an overview of participant characteristics.

**Table 1:** Description of participants (*n* = 9)

**Gender**	7 women, 2 men, 0 non-binary
Occupation	3 nurses 3 child and adolescent psychiatrists 3 heads of CAP inpatient units
Age (years)	*M* = 47.1; Mdn = 48; Range = 34–63
Clinical experience inpatient CAP (years)	*M* = 13.4; Mdn = 15; Range = 1.5–27
Region of work	6 Non-metropolitan region 3 Metropolitan region

*M* Mean, *Mdn* Median, *CAP* Child and adolescent psychiatry

### Data collection

Individual interviews with participants were 73–110 min long (Mdn = 87 min) with a total audio recording time of 13 h and 28 min. AM (at the time a late-stage child and adolescent psychiatry resident) conducted the interviews in Swedish during May–August, 2021. No follow-up interviews with participants were conducted, and only AM and the individual participant was present during each interview. AM took field notes during and immediately after the interviews. Only the audio from the video interviews was recorded and transcribed verbatim by AM. Two participants were previously acquainted with AM through working in the same organisation. All participants were informed of AM’s interest in coercive measures and related legislation, her involvement in the research group, and her residency status. No further information about AM’s personal views on coercive measures or legislation was disclosed. The interviews partly addressed the impact of a newly introduced (July 1, 2020) stricter legislation regarding coercive measure use in Sweden. We analysed these data separately and presented them elsewhere [25].

The interview guide was initially tested through a pilot interview with a child and adolescent psychiatry resident (not included in the study), after which it was revised for clarity. Information from a second planned pilot interview was included in the study, as feedback from that participant only led to minor changes in the wording of the guide. The interview guide (available in the appendix) was constructed to assess practices of compulsory care and coercive measure use, professionals’ comprehension of the changed legislation, and the use of informal coercion. To examine staff approaches to various complex patient behaviours, we constructed and used a fictional patient scenario (available in the appendix) featuring aggressive conduct, severe self-harm, and medication refusal based on clinical experience of the authors. We created the interview guide based on different theoretical frameworks, encompassing the Swedish legal framework, an implementation outcomes framework (reported elsewhere [25]) and Szmukler and Appelbaum’s suggested hierarchy of informal coercion [13]. In this study, we use the term “informal coercion” rather than “treatment pressures” to reflect the inherently coercive nature of inpatient child and adolescent psychiatric care and the potentially heightened vulnerability of young patients to various forms of coercion. Our use of “informal coercion” focuses on this spectrum of practices that may affect patient autonomy, regardless of whether they are perceived as coercive by staff or patients or not.

### Data analysis

Interview data were analysed using reflexive thematic analysis [27, 28], a method for examining qualitative data that involves a reflexive and transparent approach by the researcher to develop meaningful themes. It includes six phases: 1) familiarizing with the data, 2) generating initial codes, 3) generating initial themes, 4) reviewing themes, 5) defining and naming themes, and 6) producing the report.

An example of the coding process and theme development:

Interviewer: “Something that has also been ongoing is the COVID-19 pandemic, which started even before the legal change and is still ongoing. How would you say it has affected your work, and the care provided in the ward?”

Nurse: “Well, we have seen quite a significant increase in patients with worsening conditions, not at the beginning of COVID, and not in the first half-year, but now. When people are returning to school, many haven’t managed with school during the past year, parents have lost their jobs, there is financial hardship, and families are in crisis. For us, this means dealing with more difficult and severely ill patients, where the entire system is failing. Otherwise, we work a lot with networks, with parents to help them provide support, security, routines, and structure, but that has collapsed. So, COVID-19 has really undermined those families where the situation is most difficult. In cases where the parents have been affected — financially, losing their jobs, and with children at home more, spending more time together — it hasn’t been good for many children with mental health issues. So, for us, it’s coming to a head now; we also work very closely with the emergency department, being in the same unit, and there has been a huge increase in the number of families seeking help.

And it increased, I mean, we are now fully occupied all the time; we previously have never had a situation like this since I started here in 2015, but now there are never any available places. So now we have to prioritize discharging fairly ill patients to make room for even more severely ill patients, and that feels sad, very sad. That it has come to this. With the number of beds we have, we end up discharging patients who are still not well, which we are not used to. They used to be as stable as possible and ready to be discharged into a secure network, but that is not the case now. We are discharging them into unsafe, uncertain situations. The social services are failing on their part; there aren’t enough resources. My impression — and I have no proof for this, just my experience — is that many children are falling between the cracks and suffering, and many come back to us, and we see that things have gone badly. And that feels very disheartening.”

We initially coded this passage as *Covid: sicker patients, Covid: more problems for families, Covid: substantial increase in patients, Covid: fully occupied, increased pressure, Covid: discharging ill patients,* and *Covid: ethical stress, lower quality of care*. While the preliminary theme was labelled “Covid”, it was later revised to “Available resources and strain” to encompass broader effects on the wards beyond those specifically related to the Covid pandemic.

Under the supervision of AR, AM alone conducted a complete inductive coding of the interviews, using Microsoft Excel (version 365). Upon the initial analysis, we decided to report the data involving the potential impacts of the legal change separately. Further, given the paucity of evidence on the subject from inpatient CAP, data regarding informal coercion were deductively analysed using previously described hierarchies and theoretical models for informal coercion [13, 15, 29]. Coding and theme development were iterative processes, with findings regularly discussed among the author group to refine themes and ensure consistency.

Participant quotes included in the manuscript were translated using ChatGPT. To ensure accuracy and preserve the original nuances, AM carefully reviewed both the initial statements and their translations.

### Lived experience involvement and research team reflexivity

The study findings were discussed comprehensively with a young adult with prior lived experience of compulsory inpatient CAP care and coercive measures. She was recruited through outreach on social media in collaboration with a former lived experience council. Based on this discussion, AM drafted the lived experience commentary in Swedish, which was then translated into English, reviewed, and approved by the expert.

Reflexive thematic analysis acknowledges the active role of the researcher in developing themes, recognising that analysis is shaped by the researcher’s perspectives and interpretations. Throughout the process, we engaged in reflexivity by reflecting on our assumptions and how these may have influenced the analysis. NL (male), AM (female), SB (female) and RK (female) are child and adolescent psychiatrists and AM, RK (and formerly SB) currently serve as consultants at inpatient CAP units and have authorised the use of coercive measures for patients. AR (male) is a clinical psychologist who previously worked in adult psychiatric care. This study is part of a larger research project on inpatient CAP, with a specific focus on compulsory care and the use of coercive measures. Additional, partly related research areas, encompass risk factors for, and assessment and treatment of antisocial behaviour in adolescents (NL, RK), and patient-controlled admissions to psychiatric inpatient care (AR).

### Ethical considerations

We submitted a consultative ethics application to the Swedish Ethical Review Authority, which determined that the study did not require an ethical permit as it did not involve the collection or management of any personal records (dnr: 2020–06898). Written and verbal informed consent were obtained from all participants before the interviews. The study is reported following the Consolidated Criteria for Reporting Qualitative Research (COREQ) [30].

## Results

**Fig. 1:**
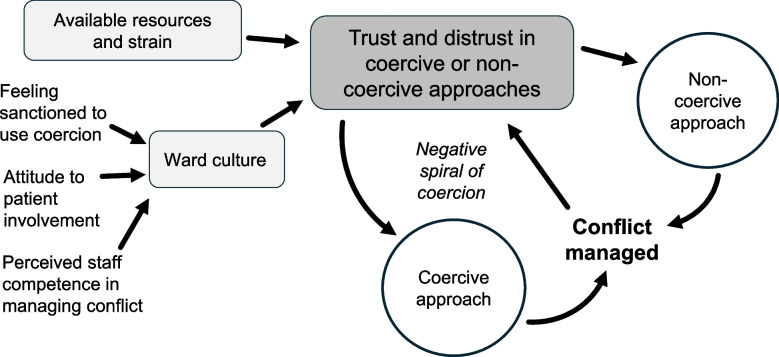
Reported aspects influencing the use of coercion/non-coercion in inpatient CAP based on staff interviews

Addressing aspects reportedly influencing the use of coercion, we identified the theme *Trust and distrust in coercive or non-coercive approaches*, with the subthemes *Ward culture*, and *Available resources and strain*. See Fig. 1 for a synthesised view of how different aspects possibly influence the use of coercion or non-coercion. Data on informal coercion were described separately using previously proposed hierarchies of informal coercion, as detailed in the Background.

### Trust and distrust in coercive or non-coercive approaches

This theme addresses different care aspects affecting trust and distrust in using coercion or non-coercion when managing conflicts at the ward. Trust or distrust in these strategies appeared to shape staff decision-making, with those expressing greater trust in non-coercive methods being more likely to use theses as first-line approaches, while staff with less trust more frequently relied on coercion. These attitudes were influenced not only by individual beliefs but also by broader structural and cultural factors at the ward level, as described in the two subthemes *Ward culture* and *Available resources and strain*.

All participants perceived coercive measures as effective for solving exceedingly difficult acute situations with patients. They considered coercive measures justified in managing aggressive and violent patients, or severely self-harming patients, who were otherwise sometimes difficult to manage safely. A head of unit stated:

“It’s the last resort as far as possible. Of course, one should appeal to voluntary participation and try to find solutions if possible to avoid them. Sometimes we end up in situations where we have to; there’s simply no other way.”

Mechanical restraint was considered highly effective in securely containing patients and ensuring unit safety during dangerous situations. While some participants approved of seclusion as a safety measure, others found it unhelpful in managing patient behaviour, particularly when designated safe seclusion rooms were lacking. Some participants also viewed coercive measures as therapeutic: with tube-feeding and forced pharmacological injections aiding clinical recovery; seclusions and forced sedative injections soothing the patient; and physical and mechanical restraint establishing “behavioural boundaries” and calming the patients.

However, coercive measures were generally seen as a failure for both the care system and staff, with some participants viewing them as such even when deemed necessary for patient care and management. A nurse said:

“It’s always a failure on our part to have to use coercive measures. That means somewhere along the way we haven’t succeeded earlier, of course. So, I can’t say there’s anything positive about it other than, if there’s no other way out, then you have to do it in the best possible way.”

Coercive measures were also considered by some participants as the “easy way out” compared to using non-coercive strategies, which were seen as requiring more effort from staff. In contrast, a few participants described coercive measures as something very rarely used, but highly necessary when used and then perceived as positive for the patient.

Great trust in non-coercive approaches was expressed by some participants, who advocated their use as a first choice and highlighted their general effectiveness. In response to the fictional case of managing a psychotic youth with violent behaviour who had smashed a chair and was armed with the leg of the chair, a head of unit described:

“If it’s a patient I know well, I would probably back out of the room and sit down—now, this may sound completely bizarre, but that’s likely what I would do first to see if we can change the situation, if we can change the patient’s experience. Because what I think is that they feel extremely cornered, pressured and that there is a lot of fear involved; that is what I would assume when I know so little.”

In contrast, other participants saw non-coercive approaches as ideal but appeared to have low trust in their effectiveness and reported using coercive measures often at their units. A consultant described the response to the same fictional patient scenario:

“I would of course press the alarm for help […], and we would make sure there were enough people… the question is how we would remove the weapon; that always makes it much, much more difficult. […] but there are two scenarios: getting people there and then […] overpowering the patient, bringing them down to the floor, and then disarming them, if possible. But when there’s a weapon involved, we are also quite quick to press the police alarm, and it’s precisely in such situations where it’s used, when there’s a weapon involved, because that makes it extremely difficult. […] It would probably be more about administering an injection right there and then, and then trying to seclude the patient.”

When opting to use coercive measures, participants described the difficult balance between creating an immediate, potentially traumatic situation and preventing greater harm in the near future. For instance, while forcibly administering intramuscular injections could erode the patient’s trust in psychiatry, it was seen as ultimately necessary for their clinical recovery. Although aware of the risk of inducing acute or post-traumatic stress-related disorders, participants generally believed that immediate care needs should take precedence.

Further, participants also stressed that using coercive measures (in contrast to non-coercive strategies) did not help patients develop new skills in demanding situations, a head of unit stated:

“The idea is that the patient should be able to manage and survive with their anxiety, but instead, it’s like you’re waiting and just dealing with the anxiety when it comes [using coercive measures] rather than working on it.”

Most interviewees advocated using coercive measures as a last option, emphasising the importance of exhausting voluntary options first and applying coercion only when absolutely necessary. Nonetheless, some participants stressed using coercive measures promptly to prevent escalation and harm to patients, staff, and property. Delaying the intervention was viewed as potentially harmful for the patient and unethical, especially regarding involuntary medication for severely ill patients with psychosis. Conversely, others argued against resorting to coercive measures too quickly, preferring to tolerate property damage rather than using force.

Some participants reported that once coercive measures were applied to a patient, it became increasingly difficult to discontinue their use, indicating a possible *negative spiral of coercion* for both patient and staff. Two heads of units independently described:

“One time might be okay, but not a second time, because then it’s like, it’s hard to turn back once you’ve started. Twice becomes a habit, you know.”

“It must not become a habit to use coercive measures, not for the staff but also not for the patient to be subjected to coercive measures. That must not become a habit either, because I think that is absolutely negative.”

In cases where patients were perceived as particularly challenging to manage, coercive approaches were often seen as inevitable, resulting in some individuals being subjected to multiple interventions. Several participants expressed a sense of resignation about using coercive measures, feeling forced to do so to manage acute situations, even when they believed it was not in the patient’s best interest. Also, some participants observed that certain patients seemed to deliberately provoke the use of coercive measures, either as a form of self-harm or to manage anxiety. This left some staff feeling powerless and forced to use coercive measures when confronted with escalating patient behaviour.

### Ward culture

This subtheme captures various aspects of ward culture affecting trust or distrust in coercive practices, including staff feeling sanctioned to use coercion, perceived staff competence to manage conflict, and attitudes to patient involvement in care.

All participants expressed a strong sense of responsibility for their patients’ well-being. They explicitly aimed to prevent harm, uphold the law, maintain patient dignity, and foster trust in psychiatric care. Participants saw their authority over patients as sanctioned by parents, the care system, the legal system (in compulsory care), and their role as adults caring for children. In addition to feeling responsible for the child’s well-being, participants also described a sense of responsibility for preserving the parent–child relationship. Staff sometimes assumed the”bad guy” role to protect parents from being seen as enforcers of coercion, aiming to maintain trust between the child and their family. In severe conflict situations, coercive measures were seen as essential to protect the patient and fellow staff. This combination of factors appeared to make participants *feel sanctioned to use coercion* if necessary: both formally authorised and informally validated. Those with experience from working in adult psychiatry noted more frequent use of coercion in the care for children and adolescents, particularly rapid responses to self-harm as compared to slower action against violence. This was attributed to a higher perceived urgency in preventing self-harm and a hesitancy to coerce violent children: attitudes that appeared to be rooted in ward culture and shaped by collective staff beliefs about appropriate responses in child/youth care.

Participants had varied *attitudes towards patient involvement in care*. Most highlighted efforts to collaborate with patients, ensuring some control over their own care also when compulsorily treated. This included encouraging patient participation in care planning and adapting to their needs. Some argued for empowering the patients and prioritising their opinions, even when these conflicted with the care team’s views. There was a strong emphasis on understanding the patient and his/her behaviour, with some participants wishing for a greater focus on understanding the motivations behind non-cooperation.

Coercive measures were perceived to clash with a caring perspective and negatively impact patient autonomy, though some staff tried to involve patients even during coercion, such as asking about their preferences when under restraint. One participant noted that in what was described as dysfunctional wards, staff could become cynical and lose curiosity in understanding the patient. Two participants stated that when staff made mistakes, they were encouraged to apologise to the patient and repair the relationship. In contrast, a few participants believed that staff knew what was best for patients and should disregard conflicting patient opinions. Some participants described rarely giving power to the patient regarding their care and stressed the importance of not questioning decisions in front of the patient, even if they personally disagreed with them.

The level of *perceived staff competence in managing conflict* varied. Most participants considered risk situations as preventable or at least foreseeable, emphasising the importance of staff interventions such as de-escalation and diversion to avoid harm and coercion. Most participants believed experienced staff to be able to professionally manage conflicts, de-escalate situations when possible, and if necessary, use coercion effectively and respectfully. As stated by a nurse:

“But then, when it starts, okay, now a situation is beginning to escalate, there is still a lot that can be done before needing to resort to a coercive measure. [...] Especially if you start with a low-arousal approach, talk the patient down, and offer medication, and so on. In my experience, this works nine times out of ten, or 99 times out of 100, if you have well-trained staff, a confident team, a good team leader, and confident doctors, along with routines in place.”

Some expressed confidence in their colleagues’ ability to prevent and manage acute situations, ensuring voluntary patient cooperation. They trusted that inexperienced staff would receive support from experienced colleagues during a conflict. However, due to a shortage of experienced staff, other participants described a lack of trust in the ward’s overall competence to manage conflicts.

Strategies for dealing with patient conflict varied. Some participants reported a clear ward strategy in dealing with patient behaviour that threatens the safety of the patient or others, always making active choices to engage in conflict or not, weighing in the risk of escalation and coercive measure use. Others described a more reactive, unstructured response to conflicts, risking quick escalation to coercion. Several interview subjects expressed a desire to work more preventively and noted that coercive measures had become routine, with insufficient efforts to provide alternative, non-coercive strategies.

### Available resources and strain

**Fig. 2:**
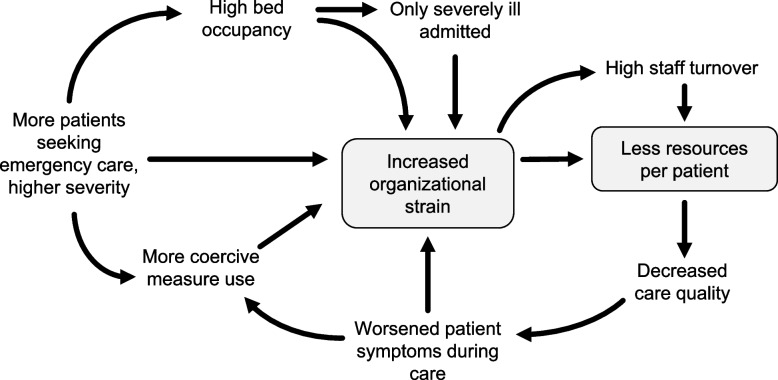
Proposed model for increased strain on inpatient CAP units based on staff interviews

This subtheme describes how the care was negatively affected by participant-reported increased work strain on the wards, paired with fewer resources. See Fig. 2 for a proposed model of the subtheme.

All participants reported substantial increases in patients seeking psychiatric emergency care and requiring hospitalisation, leading to high bed occupancy. In particular, involuntarily hospitalised patients with severe restrictive eating disorders had increased over the last years. Stricter requirements for admission following the shortage of beds resulted in admissions of only the most severely ill, mostly compulsorily treated patients being admitted, leaving few beds available for voluntary patients.

High bed occupancy and increased severity of patient morbidity were described to negatively affect care quality. Participants perceived the wards as becoming more dysfunctional, with staff struggling to properly manage patients’ psychiatric symptoms, leading to worsened symptoms among patients that the staff had previously been able to manage. Some participants noted more frequent use of coercive measures, with incidents of violence, threats, and self-harm becoming more common. A senior consultant described:

“What I can see is a worrying trend with more admissions under compulsory psychiatric care, increased pressure, and a minor system collapse that risks undermining the protective factors that have previously been important for preventing the need for coercive measures. […] because the situations have become more strained, and the staffing shortages are becoming more troubling, with experienced staff leaving, and so on.”

Coercive measure use was perceived as a negative, stressful, and potentially traumatising experience for both patients and staff. Interviewees linked coercive measure use to increased staff sick leave or job resignation and highlighted the risk that they would be used as punishment and their detrimental impact on patient-staff relationships.

As described above, participants highlighted that increased strain on the wards was compounded by insufficient resources. Most participants reported high staff turnover with difficulties to attract and keep experienced personnel, further reducing care quality. However, one head of unit described a well-functioning ward with many experienced personnel and low staff turnover:

“Staffing, staffing, staffing, and then we also prevent a lot of coercive measures. […] And the young people go to bed around ten or eleven, and then it’s pretty quiet and calm, except if someone is really struggling, in which case there are two people who can take care of them, find other ways to handle it. If they need three, then there are three; if they need four, then there are four. This winter, they needed five, so they got five. I mean, it costs money to run inpatient care, but it also costs to provide good care.”

Participants reported that the Covid-19 pandemic impacted noticeably on the wards, initially leading to a decrease in patients seeking inpatient care. However, by late 2020 and early 2021, admission rates increased again substantially; patients were then often more severely ill than before. Participants attributed this to reduced access to outpatient care, missed or cancelled appointments, or an inability to engage in digital consultations (many outpatient meetings were scheduled as digital meetings). The pandemic’s broader societal effects, particularly on socioeconomically disadvantaged families, were also seen as contributors to increased strain on psychiatric services.

### Using informal coercion

Overall, informal coercion was primarily used to ensure adherence to treatment deemed to be in the patient’s best interest. Participants justified its use when they believed the care team knew better than the patient. Strategies for handling non-cooperative patients varied according to at least two different processes. Some participants maintained a consistent level of treatment pressure, mostly with repeated persuasion efforts over time to wear down patient resistance to intervention. Others employed a stepped approach, gradually increasing pressure until the patient complied with the least amount of coercion necessary.

We found that all participants employed *persuasion*: some aimed to decide on the patient’s behalf, while others tried to motivate patients to accept voluntary care by discussing the pros and cons and encouraging ongoing treatment. A consultant stated:

“I believe more in this kind of flexibility and thinking about what we can let this young person ‘win’ over, sort of like negotiating, and I often talk a lot about compromising and trying to explain what that word means, that you can’t always get everything you want, like ‘you’re thinking that you should get this, but you can’t have it all, but would you take half? If we try to meet halfway, where would we end up?’ and so on.”

However, some participants emphasised that in acute states of mental illness, attempts at negotiating or persuading waste precious time and can be counterproductive, as patients are rarely responsive during these periods. Others described extended efforts to obtain voluntary treatment agreements.

*Interpersonal leverage* seemed to be used rarely. Some participants reported *coercion by others* as frequently exercised*,* usually by parents, while others intentionally avoided involving parents in treatment adherence, allowing the care staff to take on the role of the “bad” enforcer and leaving the parents as the supportive “good” figures.

*Inducements* seemed to be perceived as less problematic than treatment pressures lower down in the informal coercion hierarchy [13]. Participants described efforts to find motivating factors; for instance, offering children a soft drink, ice cream or sweets as a positive enforcer to take their medication, combining them with persuasion. A nurse said:

“Yeah, but you do that sometimes anyway, like, ‘come on, just take your medicine and then we’ll go play ping pong’ – it can be the simplest thing in the world. Or like, yeah, you'll get your favourite soft drink or something, yeah absolutely, you do that.”

All participants frowned upon direct approaches to *tricking* patients into treatment adherence, such as putting medicine in their drink or food without informing them. Occasionally, parents requested staff assistance to trick the child into taking medication, but participants reported not aiding with this. Participants describe the acceptability of such deception to vary with ward culture. Most participants, however, found *strategic dishonesty* more acceptable. This involved withholding certain information, such as not presenting available options or pretending options existed when they did not. Some also described not informing patients about changes in types of medication or dosages to prevent failure in treatment adherence.

*Threats* appeared to be the least approved form of informal coercion, with most participants believing threats should not be part of a care culture. However, some perceived threats being frequently used while others observed them being employed by certain staff, particularly when staff were tired. Participants generally agreed that threats were ineffective in improving patient outcomes. However, some emphasised perceiving threats as less invasive and traumatic than formal coercive measures, such as threatening to force-feed patients with restrictive eating disorders if they did not eat voluntarily.

All participants found it difficult to distinguish between informing patients of the consequences of non-adherence and threatening them with formal coercion, creating an ethical conflict about how to inform patients *without* threatening them. For example, some saw informing a patient about the necessity of an involuntary injection if they refused oral medication as a definite threat with one senior consultant reporting it as a coercive measure. Others did not interpret it as a threat given that the intention was to inform the patient. A senior consultant said:

“Because there comes a point where you have to, like, either talk about it in advance or it hits like a bolt from the blue.”

Some participants described *referring to rules and routines* to manage patient behaviour, as well as using a *disciplinary style* to get the patient to adhere to ward rules.

## Discussion

We interviewed staff across Swedish inpatient CAP services and found that trust in coercion or non-coercion appeared to influence the use of coercive approaches, along with a reliance on informal coercion in clinical practice. Our findings suggest that trust encompasses both staff beliefs in the effectiveness of coercive and non-coercive strategies and their confidence — individually and collectively — in managing conflict in a safe and patient-centred way. We propose the existence of coercion processes in the use of both formal and informal coercion: with a hypothesised negative spiral of increasing coercion, and an informal coercion process with either repeated reliance on the same level of coercion or a tiered approach.

Staff dependence on coercion may be understandable based on our findings of a strong staff sense of responsibility for the patient; combined with trusting coercion to keep the patient safe, alongside limited resources and low trust in non-coercive alternatives. This reliance on coercion within the safety paradigm reflects a perceived lack of viable alternatives in acute situations, aligning with previous research of the safety paradigm’s role in coercion use and the prioritisation of patient safety in adult psychiatry settings [18, 31]. While evidence on a direct relationship between staff experience and reduced use of coercion is inconclusive [18], adult psychiatry staff perspectives suggest that a combination of experience, effective de-escalation skills, and sufficient resources may better equip teams to implement non-coercive interventions [31].

Further, our findings emphasising trust in managing approaches agree with an integrative review of decision-making regarding coercive practices in adult psychiatry; that greater trust in staff team skills may enable more prolonged efforts at de-escalation before resorting to coercive measures [32]. However, our findings contrast with a quantitative questionnaire study of adult psychiatry staff’s implicit and explicit attitudes toward coercion [33]. These authors found no associations between attitudes and coercion use at the overall clinic level, although results might have differed if data had been analysed unit-wise. Our findings on the reported impact of different ward cultures suggest that obtaining and examining more fine-grained data is important.

The negative spiral of increasing coercion suggested here may contribute to understanding why some units seem to get caught in more coercive care practices while others successfully adopt non-coercive approaches. This negative spiral implies that once coercive measures are used by a staff constellation, the *individual patient* is at risk of “being stuck” in this approach, influenced by both staff and patient beliefs about the effectiveness of coercion or non-coercion in managing situations. Further, based on prior staff experiences of coercive approaches, *subsequent inpatients* in conflict with staff could also risk being coercively managed. This aligns with our recent systematic review suggesting both extensive variability in coercive measure use across CAP settings and that a small inpatient subgroup is frequently exposed to coercive measures [2]. The latter is also supported by prior adult psychiatry studies [34–37] and the newly proposed “Maintenance Model of Restrictive Practices” [38]. This model posits that restrictive practices are maintained through a self-sustaining cycle involving patient behaviours being perceived as dangerous, staff emotional responses (such as feelings of threat and distress), and coercive measure use being reinforced by creating a sense of safety among staff. The model also highlights the importance of contextual factors, including past traumatic experiences, power imbalances, and broader organisational influences such as leadership, policy clarity, staff training, and the physical and sensory ward environment [38]. Our own more decision-focused lens provides a complementary insight into how coercion processes unfold within the everyday clinical environment, adding granularity to the broader systemic factors described by the Maintenance Model of Restrictive Practices.

One can hypothesise that a corresponding *positive spiral of non-coercion* could also exist. Hence, if patients experience and staff employ non-coercive methods with positive outcomes, they could be more likely to respond positively to or use non-coercion in future challenging situations, with the same patient or others. Staff beliefs in their efficacy could, at least partly, contribute to why targeted programs with non-coercive approaches might be successful. We suggest that strengthened trust in non-coercive approaches could be achieved by interventions fostering (perceived) competence to handle conflict, reduced views that the use of coercion is sanctioned, and increased patient involvement. Importantly, sufficient and qualified resources and reduced strain on inpatient services appear vital to maintaining non-coercive approaches, highlighting the need for both individual- and structural-level interventions.

Coercion as a process has previously been defined from a patient perspective, a systematic review indicated that adult psychiatric patients subjected to coercive measures changed their interactions with staff out of fear of being subjected to coercive measures again [39]. This is in line with the idea of a “coercion shadow” in psychiatry [40]; with the knowledge of the risk of coercive treatment as an alternative, the patient obliges to avoid formal coercion. However, such a choice can also reflect self-determination; that the patient is actively making decisions and managing his/her behaviour. This capacity for autonomous decision-making could also be viewed as a positive outcome, indicating retained agency.

The conceptualisation of a threat as an intentional prediction of negative consequences to influence behaviour, while a warning conveys possible outcomes without an intent to harm [13, 41], may be clear in a hypothetical scenario. However, the distinction is often more blurred in clinical settings, as patients may perceive a warning as coercive. Consistent with findings from adult psychiatry [15], our results underscore this fine line between informing patients about the consequences of their actions and threatening them.

The use of informal coercion in inpatient CAP is largely unknown but our data indicate similarities with findings from adult psychiatry and mostly support previously described hierarchies and theoretical models [13, 15, 29]. However, interestingly, the use of strategic dishonesty seems more accepted, perhaps due to an inherently paternalistic viewpoint towards children; withholding information or using strategic dishonesty might be more accepted with the argument of protecting the child’s best interests. Our findings suggest that staff seldom used interpersonal leverage. However, it might still be applied, for example by parents, without staff knowledge. In line with findings from adult psychiatry [15], small inducements were considered unproblematic and often combined with persuasion. However, depending on the maturity of the child, different inducements could render independent decisions difficult. Participants seemed to perceive inducements as less problematic than persuasion, in contrast to the originally proposed coercion hierarchy; a possible explanation could be that small inducements were seen as supportive rather than coercive, particularly when framed as encouraging cooperation rather than enforcing compliance. The present findings regarding the continued use of the same level of informal coercion as a strategy to wear down resistance to non-adherence have not been described elsewhere and may be more specific to the inpatient treatment of children and adolescents.

This study focused on staff perceptions and did not explore patients’ reports of coercive experiences. Hempeler and colleagues [14] proposed a context-sensitive definition of informal coercion, where service users have a justified belief that they will face negative consequences if they do not follow caregiver recommendations. An important contextual factor in this model is patient dependency on caregivers, which may be more pronounced in CAP than in adult psychiatry, as children and adolescents typically rely more heavily on adults for care, support, and decision-making. This heightened dependency could increase their vulnerability to experiencing treatment pressures as coercive. In contrast, children may be more accustomed to being coerced and compliant with many societal norms (e.g., not being aggressive despite frustration, attending school, doing homework, obeying parental and family rules etc.) and then possibly be less sensitive to experience coercion. However, a previous study from Norwegian inpatient CAP reported that one-third of patients perceived high levels of coercion, similar to levels reported from Norwegian adult psychiatry [42]. Further research is needed to understand the patient coercive experiences in CAP compared to adult psychiatry, alongside continued investigation of staff perceptions and decision-making.

### Lived experience commentary

In contrast to the staff-based interview findings, our expert with lived experience (who prefers to remain anonymous) described that it was often the most experienced staff who routinely used coercive measures, sometimes even as punishment or discipline. New staff members attempted to build relationships and engage with patients, but many did not stay long, as the ward culture often clashed with their values. Negative aspects of the ward culture included an “us vs. them” attitude between staff and patients, a lack of patient involvement and insufficient explanations of decisions regarding their own care. As the expert put it: “It didn’t feel like they treated me as a human being, more like an object.”

The expert also shared experiences from when the ward was overcrowded; how resource shortages and overall strain on the system often affected patients. When staff appeared more stressed, this led her to avoid seeking help from trusted staff members to not add to their burden. When the ward was overcrowded, parents were not allowed to stay overnight, which increased stress for patients, who often relied on their parents for support and comfort. She also described having guilt feelings when staff implied that she was “taking up space” that could have been used for someone in greater need.

Regarding informal coercion, the expert described the harmful effects of threats, such as being threatened with mechanical restraint or being denied visits from loved ones if treatment rules were not followed. She also recounted experiences of being deceived by staff, where promises were made to encourage compliance but were not fulfilled. This strategic dishonesty, combined with the absence of promised rewards, led her to lose trust in the staff.

The difficulty of distinguishing between threats and information, which was raised by some staff participants, was seen as less problematic by the expert. She believed the difference was usually clear and could be discerned from the staff’s tone and way of expression.

The expert also emphasised the importance of not using parents as tools to enforce coercion. When parents were placed in such a role, it created fractures in the relationship, and parents were perceived as threatening rather than as a source of safety. She argued that coercion should be carried out by staff, while parents should provide support and a sense of security.

Creating a positive spiral of non-coercive care was seen by the expert as possible, particularly for patients. She reflected that if she had been met with a more respectful and inclusive approach and allowed to participate in her care, she might have received help earlier and prevented her problems from becoming so severe. This could also have made the ward a safe place to turn to in times of need.

### Strengths and limitations

This is one of the first studies to explore staff perspectives on coercive measures and informal coercion in inpatient CAP. Yet, there are limitations to consider.

First, the small sample size, perhaps attributed to the acute and busy nature of the inpatient clinical settings involved or potential reluctance from staff to discuss sensitive issues and clinical practices. However, three different professional roles and nationwide variations in the geographic and urbanicity of workplace settings provided a range of perspectives on coercive practices, contributing to data source triangulation [43]. While we believe the sample was sufficient to address the study aims, a larger sample could have provided more nuanced insights into differences between professional roles and the diverse range of coercive practices. Including different occupational categories captured a broader range of perspectives on decision-making surrounding coercion but might also have introduce complexity that may have limited the depth of discipline-specific experiences. Nonetheless, the diversity of views contributed to a more comprehensive understanding of the systemic and contextual factors influencing coercion use. Notably, we considered data saturation concerns not relevant, given the use of reflexive thematic analysis [44].

Second, the broad conceptualisation of coercion, spanning both formal and informal coercion, was intentional to reflect the complexity of how coercion manifests in everyday clinical settings. We recognise that this breadth may have reduced the focus on any single type of coercion, but it allowed us to capture the full spectrum of coercive dynamics relevant to clinical decision-making. Narrowing the focus in future research could enable more detailed exploration of specific coercive practices.

Third, the recruitment strategy, which relied on gatekeepers, helped streamline the recruitment process but could have led to the selection of participants holding particularly strong views, positive or negative, potentially affecting the representativeness of our sample compared to the broader inpatient CAP staff population.

Fourth, while interviews were in-depth and produced rich, detailed data on coercive practices, participants were not invited to review transcripts or provide feedback on the findings, a step that could have added to the study’s credibility.

Fifth, one single researcher (PhD student) conducted the coding process, albeit under supervision. This approach, while providing consistency and a deep understanding of the data, might still be problematic given the reliance on a single perspective. Even so, the researcher’s involvement in every stage — from recruitment and interviewing to transcription, coding and manuscript drafting — ensured considerable familiarity with the data, strengthening the trustworthiness of the findings.

Sixth, research team expertise within the clinical context and prior understanding of the research topics may present both strengths and weaknesses. While this familiarity might have enabled nuanced interpretation of participant responses it could also lead to interpretations that reflect pre-existing assumptions or expectations.

Last, the interviewer’s position as a resident in child and adolescent psychiatry during the interviews may have influenced responses, potentially affecting how they framed their experiences, or the level of detail provided.

To enhance judgments about the transferability of our findings to other settings and to support dependability and confirmability, we provide a detailed description of our analytical process in the methods section, with the interview guide available in the appendix. For details on the Swedish Compulsory Psychiatric Care Act and additional details on the Swedish inpatient CAP settings see the supplementary materials from our previous study [25].

### Future directions and clinical implications

Our findings suggest that staff may feel compelled to use coercive measures due to a perceived lack of alternatives or inadequate resources. Interventions that promote confidence in non-coercive approaches — along with proper training, support, and resources — are necessary to foster a culture shift. These clinical implications align with the *WPA Position Statement and Call to Action: Implementing Alternatives to Coercion* [7], underscoring the importance of enhancing staff competence in de-escalation strategies, promoting patient involvement, and addressing structural barriers to non-coercive care. By improving staff trust in non-coercive approaches and supporting patient-centred interventions, clinical practice could shift towards safer, more respectful care environments while reducing reliance on coercion. Future research should further explore what factors enhance this trust and how to effectively implement these practices across different settings.

The proposed *negative spiral of coercion* model presented here may help in understanding why some patients are repeatedly subjected to coercive measures, and why certain staff constellations or settings use coercive measures more than others; in turn highlighting the need for empirically informed strategies to interrupt this cycle. Further research may examine the validity of the suggested model, if informal coercion differs in CAP compared to adult inpatient settings, and factors that contribute to greater staff reliance on non-coercive practices. Also, exploring how violence or resistance from child inpatients is perceived and managed differently compared to adults, could help inform our understanding of when and how coercion is used.

Insights from lived experience highlight the need to address ward culture and staff dynamics, particularly the reliance of experienced staff on coercive measures and associated impact on patient trust and staff retention. The harmful effects of informal coercion such as threats and broken promises should also be explored, as these can undermine trust and patient well-being. Future research should focus on promoting inclusive care where patients and families are involved in decisions, helping to create a positive spiral of non-coercive care.

## Conclusions

Our study suggests that staff trust or distrust in the effectiveness of coercive and non-coercive approaches is crucial in how staff-patient conflicts are managed in inpatient CAP. A lack of trust in non-coercive methods, combined with perceived limitations in resources and alternatives, may contribute to a negative spiral of increasing reliance on coercive measures.

## Supplementary Information

Supplementary Material 1.

## Data Availability

The datasets generated in this study are not publicly accessible due to ethical restrictions. However, they could be made available to qualified users upon written request to the corresponding author.

## Abbreviation

CAP: Child and adolescent psychiatry/psychiatric
