# Supplementary material for: Perceived determinants of the use of coercion in inpatient child and adolescent psychiatry: a qualitative interview study with staff
Source: BMC Psychiatry. 2025 Mar 17;25:246. doi: 10.1186/s12888-025-06690-x (PMC11912791; doi:10.1186/s12888-025-06690-x)
Supplement: Supplementary file 1 — Supplementary Material 1. [file 12888_2025_6690_MOESM1_ESM.pdf]

Appendix to *Perceived Determinants of the Use of Coercion in Inpatient Child and Adolescent Psychiatry: A Qualitative Interview Study with Staff*

English translation of the interview guide

Introduction

Brief information about the study
Information about confidentiality
Information about recording and data handling
Information about the possibility of terminating participation
[Start recording]

Basic information about the interview subject

Age
Gender
Tell me about your work in psychiatry and child and adolescent psychiatry?

- Profession
- Period worked in psychiatry, in child and adolescent psychiatry, in child and adolescent psychiatric inpatient care?

Initial questions

What experiences do you have of using coercive measures in your work?

- What is your opinion about using coercive measures?
- What are the positive aspects of using coercive measures?
- What are the negative aspects of using coercive measures?

On July 1, 2020, the legislation regarding coercive measures for patients under the age of 18 was changed.

- How have you understood the legislative change?
- Has it affected your work in any way?
- What do you think is positive about the new legislation?
- What do you think is negative about the new legislation?

The Covid-19 pandemic has been ongoing both before and after the legislative change.

- How has the pandemic affected work and care at the unit?

    Is there any difference in discharge, length of stay, leave from the hospital, next of kin, compulsory care, coercive measures, or anything else that you can see? For example, not admitting someone with symptoms of a common cold and finding another solution?

Another legislative change in 2020 was that the Convention on the Rights of the Child became law on January 1. Have you perceived that it has affected care in any way? Can you give an example?

- Greater consideration for even younger patients' right to self-determination? More compulsory care as a result?

Questions about knowledge, support from the organization

- Have you received any training on how the legislation has changed and how it is intended to be interpreted?

  If yes, what was included in this training? What was good/less good? Was anything missing?

  If not, what do you think about having such training?

- How do you think other staff have perceived the legislative change, e.g., psychiatric aides, nurses, junior physicians, ward managers and senior consultants?
- Is there anything you would need in your work to make it work as well as possible to use the new legislation?

  For example, guidelines, support from the employer or the like?

Questions about indications for coercive measures

Now I will read to you a fictional case that I want you to reason about.

A is a 16-year-old boy who was compulsorily admitted a few days ago due to psychotic symptoms. He has delusions that there is a plot against him, he believes that others want to harm him and that fellow patients are reading his thoughts. When you arrive at the recreation room in the ward, A is talking loudly to himself. He sits down next to a fellow patient and talks loudly to her about the plot he is exposed to, wondering if she puts thoughts in his head. You see that the fellow patient becomes worried and seems to find it uncomfortable.

What would you do in such a situation?

A refuses to leave the recreation room and wants to stay with the fellow patient. What do you do now?

If he starts to undress and sits naked in the day room, what would you do then? [Illustrates how to interpret **disruptive behaviour** versus aggressive behaviour.]

- Does it differ what you would do before and after the legal change?

The fellow patient goes to her room, A is now alone in the recreation room and starts screaming at you that you want to harm him and that you should stop reading his thoughts. He takes a chair and throws it at you. He breaks off a leg of another chair and now has a relatively large wooden stick in his hand, waving it around.

What do you do now?

A refuses to let go of the wooden stick and now rushes screaming towards you with the stick raised in the air.

What do you do now? [Illustrates how to manage **aggressive behaviour**]

Does it differ what you would do before and after the legal change?

A few days later, you hear sounds from A's room as you pass by. You look through the open door and see that he is self-harming by banging his head against a sharp edge by the window. He bangs harder and harder, has a wound in his forehead and blood is running down his face. He mumbles that he must get "it" out of his head.

What do you do now?

He continues to bang harder and harder; the wound becomes deeper, and he bleeds more and more, a large pool of blood has formed on the floor.

What do you do now? [Illustrates how to manage **self-harming behaviour**.]

Does it differ what you would do before and after the legal change?

Questions about possible occurrence of informal coercion

We continue with the fictional case: *A still has psychotic delusions about the world and seems to suffer greatly from his condition. Antipsychotic medication has been prescribed for him, but he refuses to take it.*

Nurses: *You are to administer the medication to him, but he pushes the medicine cup away and says he doesn't want to take the medication.* What do you do then?

Senior consultant: *The nurse comes and tells you that he pushed away the medicine cup and says he doesn't want to take the medication.* What do you do then?

Ward manager: *At the ward round, you hear that A pushed away the medicine cup and says he doesn't want to take the medication.* What do you do then?

- There are many ways to act in such a situation: You can try to persuade in various ways - do you have any thoughts on how you might act?
- The way one acts is often influenced by how well one knows the patient - do you have any thoughts on how it affects you in such a situation?
- It is also not uncommon to try to involve parents. What do you think about that? Could it happen - how could such a situation look like? Can you elaborate?
- It could also be that you negotiate with the patient, “if you do this, then you can get this”? Have you experienced that this occurs in any way?
- Have you experienced or heard of someone in any way deceiving a patient into something?

- Sometimes you can end up in situations where you more or less threaten the patient - for example, not being allowed to participate in an activity unless you do a certain thing. Could that happen, do you think? Can you elaborate? Have you seen it used?

A has become more anxious throughout the day and talks loudly about a conspiracy and that he knows the staff wants to harm him. He still refuses to take his medication. [Asked if threats of using coercive measures have not been mentioned].

What would you do in this situation? [Addressing the possibility of threats of coercive measures if the patient does not comply voluntarily?]

This last part of the case is about patients sometimes not wanting to do what we may think is best for them. Besides what you have already described, are there any other ways you usually manage it?

Do you perceive any difference in how these situations are managed after the change in the law?

[Reminder about confidentiality]

Questions about the use of coercion and involuntary treatment

- How do you use coercion on the ward now? Does it differ from how you used coercion before the law changed? What is your experience?

    Which patients are restrained?

    Which patients are secluded?

    Which patients receive forced medication?

    Have there been any changes in other coercive measures, such as forced feeding?

    Do you perceive any changes in how long patients are restrained or secluded now?

    Do you perceive any changes in how patients are medicated now?

- Do you think the law is being followed or is the staff trying to find ways around it to adapt to difficult situations? What is your experience?
- Do you perceive any differences in how coercion is justified in the medical records compared to before the law changed?

    Differences concerning agitated patients? Hypothesis about stressing different factors?

- In your experience, are there any differences in the patients who are treated under the new law compared to before the law changed?
- **On boys and girls:** Do you think there are any differences in how coercion or involuntary treatment is thought about or used for girls and boys? If so, what are they?

[Exploring whether there are gender aspects beyond the clinical picture, symptoms, and disabilities].

Questions about the consequences of the new legislation

- We talked earlier about the possible positive and negative aspects of the new legislation. What about the consequences? Have you noticed any consequences of the changed legislation?

    Have you noticed any changes for yourself or other staff members?

    For example, increased administrative work, increased concern for violence on the ward, increased uncertainty about what is legally required?

    Have you noticed any changes for patients?

    For example, more disturbed by other patients, increased violence?

    Have you noticed any changes in injuries or the risk of injuries for staff members?

    How do you think things like working night-time and on-call have been affected?

    Has the new legislation resulted in changed costs for the organization/clinic?

    Have you noticed any other consequences that we have not discussed?

Conclusion of the interview

- Is there anything else you have thought of that we have not discussed?

[Checking in on well-being, providing information about the possibility of contacting occupational health services if needed]

[Information about confidential information, how the material will be used]
